# Association of the mean platelet volume and red cell distribution width with dipper and non-dipper blood pressure in prehypertensive non-smokers

**DOI:** 10.1186/s13104-019-4868-x

**Published:** 2019-12-23

**Authors:** Mohammadreza Taban Sadeghi, Zahra Soroureddin, Masoud Nouri-Vaskeh, Pantea Nazarpoori, Saeideh Aghayari Sheikh Neshin

**Affiliations:** 1 Cardiovascular Research Center, Tabriz University of Medical Sciences, Tabriz, Iran; 2 Faculty of Medicine, Shahid Beheshti University of Medical Sciences, Tehran, Iran; 3 Immunology Research Center, Tabriz University of Medical Sciences, Daneshgah Street, P.O. Box: 5166614766 Tabriz, Iran; 4 Faculty of Medicine, Guilan University of Medical Sciences, Rasht, Iran

**Keywords:** Blood pressure, Dipper, Mean platelet volume, Prehypertension, Red blood cell distribution width

## Abstract

**Objective:**

Absence of nocturnal blood pressure (BP) dipping is associated with poor health outcomes, including increased mortality. Non-dipper BP seems to be a predictor of cardiovascular damage in hypertensive patients. The aim of this study was to investigate the association of the mean platelet volume (MPV) and red cell distribution width (RDW) with nocturnal dipping/non dipping status in newly diagnosed and untreated prehypertensive non-smokers, using ambulatory BP monitoring.

**Results:**

Twenty-eight patients (15 males) in the dipper group and 24 patients (11 males) in the non-dipper group were evaluated in this study. The age of patients was 41.64 ± 15.01 and 37.96 ± 15.08 years in the dipper and non-dipper groups, respectively. The rate of nocturnal systolic BP drop in the dipper and non-dipper groups was 13.79 ± 3.35% (10.20–22.10) and 5.96 ± 2.87% (1.10–9.30) (P < 0.001), respectively. Also, the mean rate of nocturnal diastolic BP drop in the dipper and non-dipper groups was 17.02 ± 5.09% (10.30–26.90) and 6.19 ± 2.75% (1.20–9.70) (P < 0.001), respectively. RDW and MPV were significantly higher in non-dipper patients than dipper patients (P = 0.001 and P = 0.012, respectively). Bivariate analysis revealed that MPV was inversely correlated with the nocturnal systolic BP drop (P = 0.005, r = − 0.385). Furthermore, RDW was inversely correlated with systolic BP drop (P = 0.019, r = − 0.324).

## Introduction

Cardiovascular diseases are the major cause of morbidity and mortality worldwide [1]. Clinical evidence suggests that prehypertension increases the risk of ischemic complications. Since homeostasis may play a role in the development of hypertension, it may be also involved in the development of prehypertension. Prehypertension is described as blood pressure (BP) between 120/80 and 139/89 mmHg [2]. Based on some previous studies, prehypertension may lead to an increase in many non-lethal target organ damages, including advancement of coronary atherosclerosis [3], cerebral ischemia [4], and also coronary ischemia [4]. Moreover, it can trigger retinal vascular changes [5].

Ambulatory BP monitoring (ABPM) is a valuable tool, which can be used to measure variability and circadian changes during 24 h. It becomes an indispensible instrument to diagnose and manage people with abnormal BP [6, 7]. A nocturnal BP drop of 10% or more in systolic BP (SBP) and a small postprandial reduction of BP in comparison with daytime BP characterize “dippers” in contrast to “non-dippers” in whom a nighttime fall in BP is attenuated or absent [8]. A variant of non-dipping seems to be a predictor of cardiovascular events in patients with hypertension [9].

Red blood cell distribution width (RDW) is a convincing and inexpensive biochemical parameter, which indicates the erythrocyte volume and size. It is determined as part of complete blood cell count. The higher range of RDW indicates the augmentation of erythrocyte heterogeneity [10, 11]. RDW usually rises due to ineffective erythropoiesis or increased erythrocyte destruction [12]. It is of major importance in patients with ST-elevation myocardial infarction [13], as well as candidates for coronary angiography [14]. Changes in RDW range can potentially predict coronary artery disease and other cardiovascular disease outcomes [15, 16], including pulmonary hypertension [17] and heart failure [18].

Previous studies revealed that patients with high BP could have a higher range of RDW in comparison to patients with normal BP [11]. Furthermore, the non-dipper pattern of hypertension may represent a significantly higher RDW range, compared to patients with a dipper pattern of high BP [11]. Although there is limited evidence to investigate the relationship between RDW and dipping BP pattern, the non-dipping pattern is introduced as an independent risk factor, which plays a major role in the pathogenesis of plaque aggregation and infarction.

The mean platelet volume (MPV) is a quantitative indicator of the average size of platelets [19]. Larger platelets are enzymatically and metabolically more active and have greater thrombotic properties than smaller platelets [20]. Compared to smaller platelets, larger platelets have a higher density of collagen aggregation, higher thromboxane A2 concentration, and higher expression of receptor glycoprotein Ib and IIb/IIIa [21]. MPV is generally associated with platelet function and activation and is defined as a potential marker of cardiovascular diseases [21].

With this background in mind, the aim of this study was to investigate the association of MPV and RDW with the dipping/non-dipping pattern of nocturnal BP in non-smokers with prehypertension.

## Main text

### Patients and methods

#### Design and subjects

In this prospective cross-sectional study, newly diagnosed, untreated, prehypertensive, non-smoker patients, who were referred to the outpatient clinic of a tertiary referral hospital in Tabriz, Iran, were enrolled between February 2018 and July 2018. All patients gave an informed consent, and the study protocol was approved by the Medical Ethics Committee.

#### Study population

Prehypertension was defined as office measurements of SBP between 120 and 139 mmHg and/or diastolic BP (DBP) between 80 and 89 mmHg. Individuals with SBP ≥ 140 mmHg and/or DBP ≥ 90 mmHg were diagnosed as hypertensive [22]. The inclusion criteria were males and females above 20 years with a prehypertension status. The exclusion criteria were as follows: normo/hypertension assessed by 24-h ABPM; confirmed history of hypertension, myocardial infarction, valvular heart disease, heart failure, or peripheral vascular disease; use of antihypertensive drugs for any reason or other drugs that may influence platelet size; diabetes mellitus; renal or hepatic dysfunction; hematological disorders; history of malignancy; acute or chronic infection; and stroke.

#### BP measurements

ABPM was used as the BP recording device (Oscar-2, SunTec Medical Inc., Chino Hills, CA, USA). The device was set for 24-h measurements every 15 min during waking hours and every 90 min during sleep by patient declaration. At least a 10% reduction in nocturnal BP compared to daytime BP was defined as dipping, while no reduction in nocturnal BP compared to daytime BP was defined as non-dipping [8].

#### Laboratory analysis

Blood samples were drawn from the cubital vein via careful vein puncture in a 21-gauge, 2 mL sterile syringe without stasis at 08.00–10.00 am. Blood samples were collected in dipotassium ethylenediaminetetraacetic acid (EDTA) tubes. An automatic blood cell counter was used for whole blood cell counts. RDW and MPV were measured in blood samples collected in EDTA tubes and analyzed with an Abbott Cell-Dyn 1800 hematology analyzer (Abbott Laboratories, Chicago, IL, USA). The expected values of RDW in the laboratory ranged from 11.5 to 14.5 fL.

#### Statistical analysis

SPSS for Windows Version 17.0 (SPSS Inc., IL, USA) was used for all statistical calculations. Continuous variables are expressed as mean ± standard deviation (SD), and categorical variables are expressed as percentage. Independent t-test was used to compare MPV and RDW differences between the dipper and non-dipper groups. Correlations between variables were analyzed by Spearman’s rank correlation coefficient. P-value less than 0.05 was considered statistically significant.

### Results

Demographic characteristics, medical history, family history of cardiovascular diseases, mean day/night time BP, and day/night mean blood pressure (MBP) are shown in Table 1. A total of 52 patients in the prehypertension stage were enrolled in this study. Twenty-eight patients (15 males) were included in the dipper group and 24 patients (11 males) were included in the non-dipper group. There is no difference in gender distribution and no difference in age (P = 0.05, for both).

**Table 1:** Baseline characteristics of dipper and non-dipper groups

Variables	Dipper (N = 28)	Non-dipper (N = 24)	P value
Sex (male/female)	15 (53.6%)/13 (46.4%)	11 (45.8%)/13 (54.2%)	0.579
Age (years)	41.64 ± 15.01 (17–81)	37.96 ± 15.08 (18–71)	0.383
BMI (kg/m^2^)	24.66 ± 3.60 (16.97–31.70)	25.79 ± 3.82 (17.92–35.18)	0.285
Medical history			
Diabetes mellitus	1	2	0.374
Hyperlipidemia	2	1	
Coronary artery diseases	2	2	
Day time SBP (mmHg)	131.89 ± 4.71	130.54 ± 3.95	0.266
Day time DBP (mmHg)	85.08 ± 1.76	85.21 ± 1.66	0.786

*BMI* body mass index, *SBP* systolic blood pressure

The mean ± SD of nocturnal SBP drop in the dipper and non-dipper groups was 13.79 ± 3.35% (10.20–22.10) and 5.96 ± 2.87% (1.10–9.30) (P < 0.001), respectively. The mean rate of nocturnal DBP drop in dippers and non-dippers was 17.02 ± 5.09% (10.30–26.90) and 6.19 ± 2.75% (1.20–9.70) (P < 0.001), respectively.

**Fig. 1:**
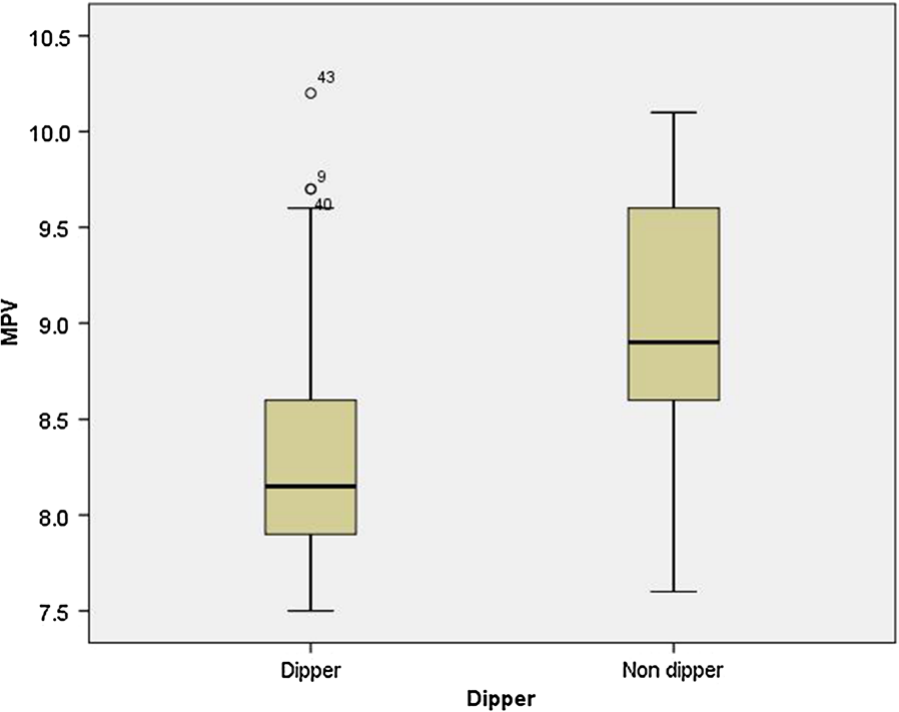
The diagram of MPV in dipper and non-dipper groups

**Fig. 2:**
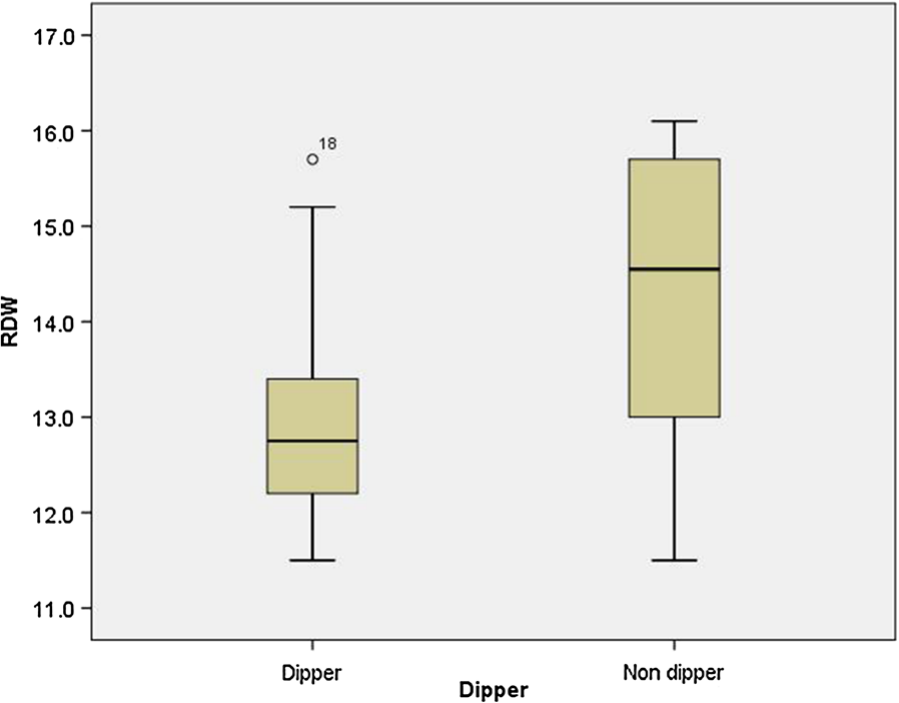
The diagram of RDW rate in dipper and non-dipper groups

Independent t-test showed that MPV in the dipper group was significantly lower than that of the non-dipper group (P = 0.012) (Fig. 1). Moreover, independent t-test showed that RDW in the dipper group was significantly lower than that of the non-dipper group (P = 0.001) (Fig. 2).

Moreover, the bivariate analysis revealed that MPV was inversely correlated with nocturnal SBP drop (P = 0.005, r = − 0.385). Also, MPV was inversely correlated with nocturnal DBP (P = 0.033, r = − 0.296). The findings showed that RDW was inversely correlated with the SBP drop (P = 0.019, r = − 0.324). Similarly, RDW was inversely correlated with the reduction of nocturnal DBP (P = 0.022, r = − 0.317).

### Discussion

Out findings revealed that dipping/non-dipping pattern in the circadian BP course is associated with elevated RDW and MPV in prehypertensive non-smoker patients. Moreover, the MPV and RDW had an inverse significant association with SBP and DBP in these patients.

Accumulating evidence asserts that prehypertensive patients are at a high risk of hypertension and that prehypertension has a linear association with the target organ damage [22]. SBP and DBP are independently predictive of target organ damage and cardiovascular events [23]. However, there are major differences in the extent of target organ damage and dipping/non dipping pattern of BP [8, 24].

Increase of BP is strongly linked to an increase in mortality of ischemic events [23]. The association between BP and mortality increases at 115/75 mmHg. Moreover, every 20-mmHg increase in SBP or a 10-mmHg increase in DBP could lead to a two-fold increment in the mortality rate [23]. Vasunta et al. [25] found an association between atherosclerosis and non-dipping hypertension and showed that high nocturnal BP induces further endothelial damage.

Different kind of pathophysiological changes including vasoconstriction, vascular wall remodeling and in situ thrombosis play a major role in inducing high blood pressure [26, 27].

Increased platelet aggregation and activation involved in the process of hypertension. The platelet evocation is important to mediating immune response and protects vascular homeostasis. Large platelets are more active metabolically and enzymatically than small platelet, and could secret more prothrombotic materials [26, 28].

Prior findings ascertained that raised MPV and RDW are an independent risk factor for myocardial infarction in patients with coronary heart disease and also predict death or recurrent vascular events after myocardial infarction [29]. MPV seems to be higher in non-dipping hypertensive patients, compared to dipping hypertensive patients [30]. In this study, MPV was higher in non-dipping prehypertensive patients, compared to dipping prehypertensive patients. Also, previous studies reported a significant increase in MPV among prehypertensive patients, which is similar to the present results [30].

RDW, an index of variability in circulating erythrocyte size [31, 32], is used for the diagnosis of anemia and is still considered a novel risk predictor of mortality [33]. However, previous trials could not provide accurate information regarding its reasonability. Gunebakmaz et al. [34] evaluated RDW among patients with high blood pressure and normal blood pressure and revealed an increase in RDW among high blood pressure groups, compared to the controls. Also, they concluded that non-dipper hypertensive patients had a higher RDW range, compared to dipper hypertensive patients. The mean SBP significantly increased at higher RDW. In this regard, Perlestein et al. found a significant increase in RDW among individuals with higher SBP [35].

RDW, as an inflammatory marker, elevates in both non-dipper and dipper patients, compared to normotensive patients [36]. There is no clear evidence explaining the pathological mechanism of higher RDW in dipper hypertension or development of hypertension, but some hypotheses have considered the effects of oxidative stress and inflammation on increased RDW among non-dippers. Generally, inflammatory cytokines can inhibit the mutation of erythroid cells by suppressing the bone marrow [37], possibly leading to the entrance of immature erythrocytes into blood circulation. Further increment of immature erythrocytes may also increase heterogeneity and result in high RDW.

Oxidative stress occurs following inflammation and may be associated with high RDW ranges; it may be expressed by oxidative stress expansion and inflammation in non-dipping patients. Increase of oxidative stress in RBCs can lead to the disruption of the mechanical properties of RBCs, which deteriorates tissue reperfusion and increases RDW [38]. In this regard, Jithesh et al., by evaluating C reactive proteins and RDW in hypertensive and normotensive patients, observed an increase in C reactive proteins and RDW of hypertensive patients, who were otherwise normal compared to normotensive cases [39].

Another explanation is the reduction of erythropoietins due to the mechanical disruption of RBCs, which activates the adrenergic system. Angiotensin II can stimulate the proliferation of erythrocyte progenitors and increase the RDW range [40]. Overactivity of the sympathetic nervous system may also increase erythropoiesis and affect the stimulation of erythropoiesis [41]. It has been also suggested that after chemical sympathectomy, exogenous norepinephrine restores erythropoiesis in rats [42].

Concern about the increased prevalence of elevated blood pressure, whereas holter monitoring is expensive device which needs to devote time and not convenient for all patients, it could be helpful to utilizing MPV and RDW in a scoring system. Moreover, it could be useful to apply it as a convenient, fast, and effective diagnostic marker in clinics.

In conclusion, RDW and MPV were significantly higher in non-dipper patients, compared to dipper prehypertensive non-smoker patients and might be used as a biomarker in scoring system in patients with the risk of cardiovascular events.

## Limitations

This cross-sectional study had some limitations. The small sample size was the major limitation of this study. Therefore, further studies with a large sample size and meta-analyses are recommended to establish the pathophysiological and clinical significance of increased MPV and RDW in patients with prehypertension.

## Data Availability

The datasets used and/or analysed during the current study are available from the corresponding author on reasonable request.

## Abbreviations

ABPM: ambulatory blood pressure monitoring
BP: blood pressure
DBP: diastolic blood pressure
EDTA: ethylene diamine tetraacetic acid
MBP: mean blood pressure
MPV: mean platelet volume
RDW: red cell distribution width
SD: standard deviation
sbp: systolic blood pressure
